# Expression of aquaporins in a transgenic mouse model of Alzheimer's disease

**DOI:** 10.1186/1743-8454-7-S1-S31

**Published:** 2010-12-15

**Authors:** Sic L Chan, Meenu Madan, Srinu Chigurupati, Jogi V Pattisapu

**Affiliations:** 1 Burnett School of Biomedical Sciences, University of Central Florida College of Medicine Room 335, Bldg 20, 4000 Central Florida Blvd, Orlando FL 32816, USA

## Background

Several studies reported altered expression of the water channels, aquaporins (AQPs), in brains of Alzheimer’s disease (AD) but the relevance of the observed changes with respect to the neuropathology of AD is poorly understood. Because aberrant processing of the amyloid precursor protein (APP) to amyloid beta-peptide (A-beta), the principle component of amyloid plaques, is central to the pathogenesis of AD, we sought to determine the temporal and spatial expression of AQPs in the cortex and hippocampus of the triple transgenic mouse model of Alzheimer’s disease (3xTg-AD) and to elucidate whether AQPs contribute directly to the aberrant processing of APP into A-beta.

## Materials and methods

We used a mutant strain 3xTg AD mice, which harbor the human presenilin1 (PS1 M146V), APP (APPSwe) and tau (tauP301L) transgenes and develop age-dependent accumulation of both plaques and tangle pathologies in a pattern consistent with AD. Cortex and hippocampus (free of choroid plexus) were harvested from 2, 6 and 14 month-old 3xTg AD and wild type (C57) mice. AQP1 and AQP4 protein expression was quantified by immunoblotting, and immunohistochemistry was performed on frozen sections probed with anti-AQP1 and anti-AQP4 antibodies. AQP1 and AQP4 mRNA expression was measured by real-time PCR.

## Results

AQP1 but not AQP4 was detected at significantly higher levels in 3xTg-AD mice at 12 months when compared with younger mice at 2 and 6 months of age. No significant changes in the expression of AQP1 and AQP4 were observed at any ages in non-transgenic control mice. Immunohistochemical analyses to determine co-localization of AQP1 with APP in neurons (in 12 month-old 3xTg-AD mice) are being undertaken. Our findings correlate with published data suggesting amyloid clearance abnormalities in chronic and normal pressure hydrocephalus.

## Conclusions

Our findings show increased expression of AQP1 in the brains of older 3xTg-AD but not non-transgenic control mice. This finding is consistent with published data of AQP1 in AD, and maybe related to similar findings in chronic or normal pressure hydrocephalus. Increased AQP1 expression in late stages of AD may possibly contribute to aberrant APP processing, and warrants further study.

